# Knowledge Graph Embeddings for ICU readmission prediction

**DOI:** 10.1186/s12911-022-02070-7

**Published:** 2023-01-19

**Authors:** Ricardo M. S. Carvalho, Daniela Oliveira, Catia Pesquita

**Affiliations:** LASIGE, Faculty of Sciences, University of Lisbon, Lisbon, Portugal

**Keywords:** Semantic annotations, Ontologies, ICU readmission prediction, Machine learning, Knowledge Graph embeddings

## Abstract

**Background:**

Intensive Care Unit (ICU) readmissions represent both a health risk for patients,with increased mortality rates and overall health deterioration, and a financial burden for healthcare facilities. As healthcare became more data-driven with the introduction of Electronic Health Records (EHR), machine learning methods have been applied to predict ICU readmission risk. However, these methods disregard the meaning and relationships of data objects and work blindly over clinical data without taking into account scientific knowledge and context. Ontologies and Knowledge Graphs can help bridge this gap between data and scientific context, as they are computational artefacts that represent the entities of a domain and their relationships to each other in a formalized way.

**Methods and results:**

We have developed an approach that enriches EHR data with semantic annotations to ontologies to build a Knowledge Graph. A patient’s ICU stay is represented by Knowledge Graph embeddings in a contextualized manner, which are used by machine learning models to predict 30-days ICU readmissions. This approach is based on several contributions: (1) an enrichment of the MIMIC-III dataset with patient-oriented annotations to various biomedical ontologies; (2) a Knowledge Graph that defines patient data with biomedical ontologies; (3) a predictive model of ICU readmission risk that uses Knowledge Graph embeddings; (4) a variant of the predictive model that targets different time points during an ICU stay. Our predictive approaches outperformed both a baseline and state-of-the-art works achieving a mean Area Under the Receiver Operating Characteristic Curve of 0.827 and an Area Under the Precision-Recall Curve of 0.691. The application of this novel approach to help clinicians decide whether a patient can be discharged has the potential to prevent the readmission of $40\%$ of Intensive Care Unit patients, without unnecessarily prolonging the stay of those who would not require it.

**Conclusion:**

The coupling of semantic annotation and Knowledge Graph embeddings affords two clear advantages: they consider scientific context and they are able to build representations of EHR information of different types in a common format. This work demonstrates the potential for impact that integrating ontologies and Knowledge Graphs into clinical machine learning applications can have.

## Background

ICU admissions are typically associated with severe disease or trauma. ICU readmissions correspond to a patient returning to the ICU after being discharged and are associated with below standard clinical outcomes, increased length of both ICU and hospital stay, and higher care costs [1]. About 1 in every 10 patients discharged from ICU units across developed countries end up being readmitted during the same hospital stay [2]. Moreover, the rate of readmission has been proposed as a marker to measure the quality of care, and it can also impact other markers such as length of stay and mortality.

The decision to release a patient from the ICU can take into account a variety of data and factors. As hospitals become more data-oriented with the increased and incentivized adoption of EHR [3, 4], we have witnessed a rise in the development of computational approaches to support clinical decision and predictive approaches [5–8]. Machine Learning (ML) has been applied to several ICU settings [9], such as predicting mortality and length of hospital stay [10], sepsis [11], mortality in diabetic patients [12], patients survival [13], cardiac arrest on sepsis patients [14], risk of acute kidney injury [15], and risk of readmission within 30 days after ICU discharge [16–18].

Despite the increasing success of ML approaches, most works still explore EHR data directly without taking into account its meaning or context. Clinical knowledge, although abundant in external sources, is not accessible to these methods, who work blindly over the data, without considering the meaning and relationships between the data objects. An example of missed context that can impair the exploration of EHR data can be seen when comparing two diagnoses: *‘Aortic Valve Disease’* and *‘Coronary Artery Disease’*. Using categorical analysis these two diagnosis have no similarity, and with a string similarity analysis they have low similarity, sharing only the less informative word *‘disease’*. However, the two diagnoses are closely related. When controlled vocabularies are used, we gain an extra layer of information given by the standardization (two entries with the same code mean the same thing) and also by the hierarchy that organizes the vocabulary. However, controlled vocabularies are limited in their contextual richness, and moreover, in a single EHR multiple domains can be covered by different controlled vocabularies which makes their concerted analysis more difficult.

**Fig. 1:**
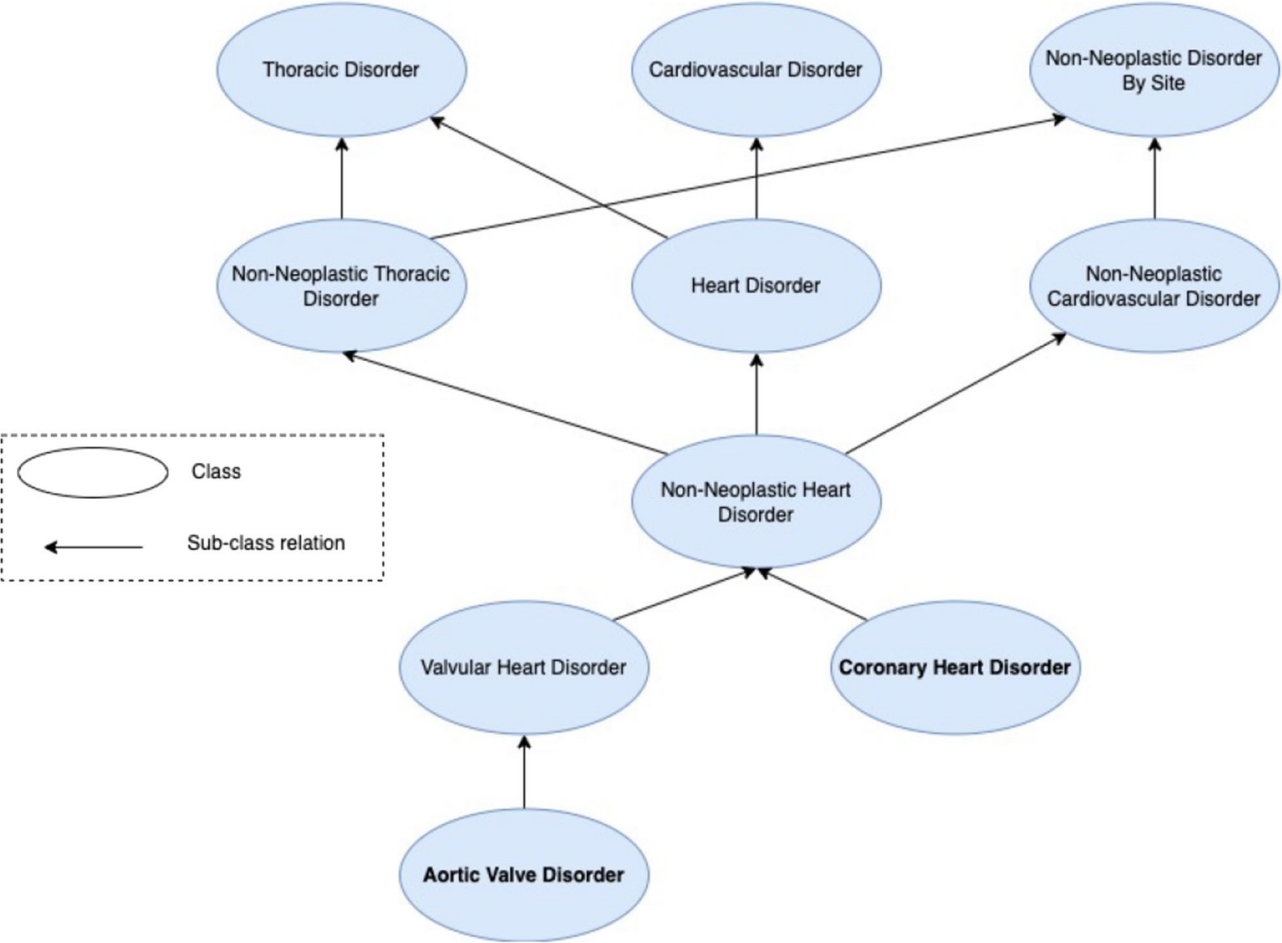
Ontology subgraph representing the classes ‘Aortic Valve Disease’ and ‘Coronary Artery Disease’

Ontologies can help bridge this gap between data and scientific context, since they are computational artefacts that represent the entities in a domain and how they relate to each other in a formalized fashion [19]. Biomedical ontologies have become quite popular in the last decades to support the annotation of the massive amounts of data produced by gene sequencing technologies. At the same time, clinical ontologies have also been developed to tackle the limitations of controlled vocabularies and allow for a fuller semantic representation. A core aspect of biomedical and clinical ontologies is that they typically encode several synonyms for the same concept, addressing the issue of synonymy. The opportunity here is that by linking EHR data to the ontologies through semantic annotations, we can feed this extra later of information about the meaning of the data to machine learning systems. Going back to the example of *‘Aortic Valve Disease’* and *‘Coronary Artery Disease’*, when these concepts are described in an ontology (e.g., Fig. 1) then it becomes evident that they are similar, since they both are non-neoplastic heart disorders, sharing after all a considerable amount of similarity as shown by their shared ancestor classes, facts that are hidden on raw analysis. An ontology can be represented as a graph, where nodes correspond to classes (that describe concepts in the domain) or individuals (the actual data entities), and edges correspond to relations between the classes and/or individuals. When a relevant portion of the graph corresponds to instances, it can be considered a Knowledge Graph (KG).

The aim of this work is then to investigate how enriching EHR data with ontology-based semantic annotations to build a KG and applying machine learning techniques that explore them can impact the prediction of ICU readmission risk, i.e., the prediction of whether a patient will be readmitted to the ICU or die within 30 days of release. We propose a novel end-to-end approach that is able to first, select the appropriate biomedical ontologies to annotate the EHR data, then use them to generate semantic representations of patients through KG embeddings based on multiple ontologies, which are finally given to ML algorithms to learn the predictive model. KG embeddings allow a seamless integration of graph-based data and vector-based data and are able to capture the semantic aspects of the KG. Other approaches, such as Graph Neural Networks expect input solely in the form of a graph, while more simple techniques such as one-hot encoding or graph-based feature extraction, struggle with capturing more complex semantics and with high-dimensionality issues. Moreover, we tackle two challenges that general applications of KG embeddings for supervised learning do not address [20], namely (1) how to select appropriate ontologies to describe the EHR data; (2) how to employ multiple ontologies to create a KG embedding based representation.

KG embeddings have been successfully employed in biomedical ML tasks such as predicting gene-disease associations [21, 22], drug-disease association prediction [23], drug-drug interaction prediction [23], protein-protein interaction prediction [23], prediction of drug-target interactions and polypharmacy side effects [24], and also in clinical applications such as miscarriage risk assessment [25] and EHR classification [17, 18, 26].

Lu et al. [17, 18] uses the discharge summaries of patients to predict ICU readimissions. In a first work [18], they employ hyperbolic embeddings of International Classification of Diseases, Version 9 (ICD9) concepts to represent the summaries to support supervised learning. In a second work [17] they represent the summaries with multi-view graphs enhanced by the Unified Medical Language System Metathesaurus, which are coupled with a Graph Convolutional neural network (CNN). These works however, are limited to a single ontology or controlled-vocabulary source and cover just one type of feature.

A parallel line of research has focused on embedding the EHR data directly, without using ontologies and KGs. Choi et al. [27] explored the natural hierarchical structure of EHR data to produce multi-level embeddings to predict heart failure, while in [28], Graph Convolutional Transformers were used to jointly learn the structure of EHR data while performing ICU readmission prediction and mortality prediction. These works do not explore existing KGs and ontologies, or lack the ability to use external sources of knowledge like ontologies, and rather build them from the EHR data, and as such do not take into consideration the context and semantics that ontologies afford.

However, most ML approaches for EHR data work over vector data and ICU readmission prediction is no exception [16, 29, 30]. Two of these works [29, 30] use unpublished data and a variety of more classical ML approaches ranging from Naive Bayes to Gradient Boosting. Lin et al. [16] however, use the publicly available dataset MIMIC-III [31] and incorporate multiple types of clinical data features and pre-trained ICD9 embeddings based on clinical notes coupled with CNN and Long short-term memory (LSTM).

Finally, there is an untapped opportunity to build models that more closely align with the reality of the ICU. To achieve this, we also aim to establish models that work with information limited to specific points in time of an ICU stay. So instead of making predictions only post ICU stay, predictions are made throughout the stay, allowing clinicians and health care practitioners to keep track of the 30-days risk of readmission and update it as more information on the patient becomes available.

## Methods

**Fig. 2:**
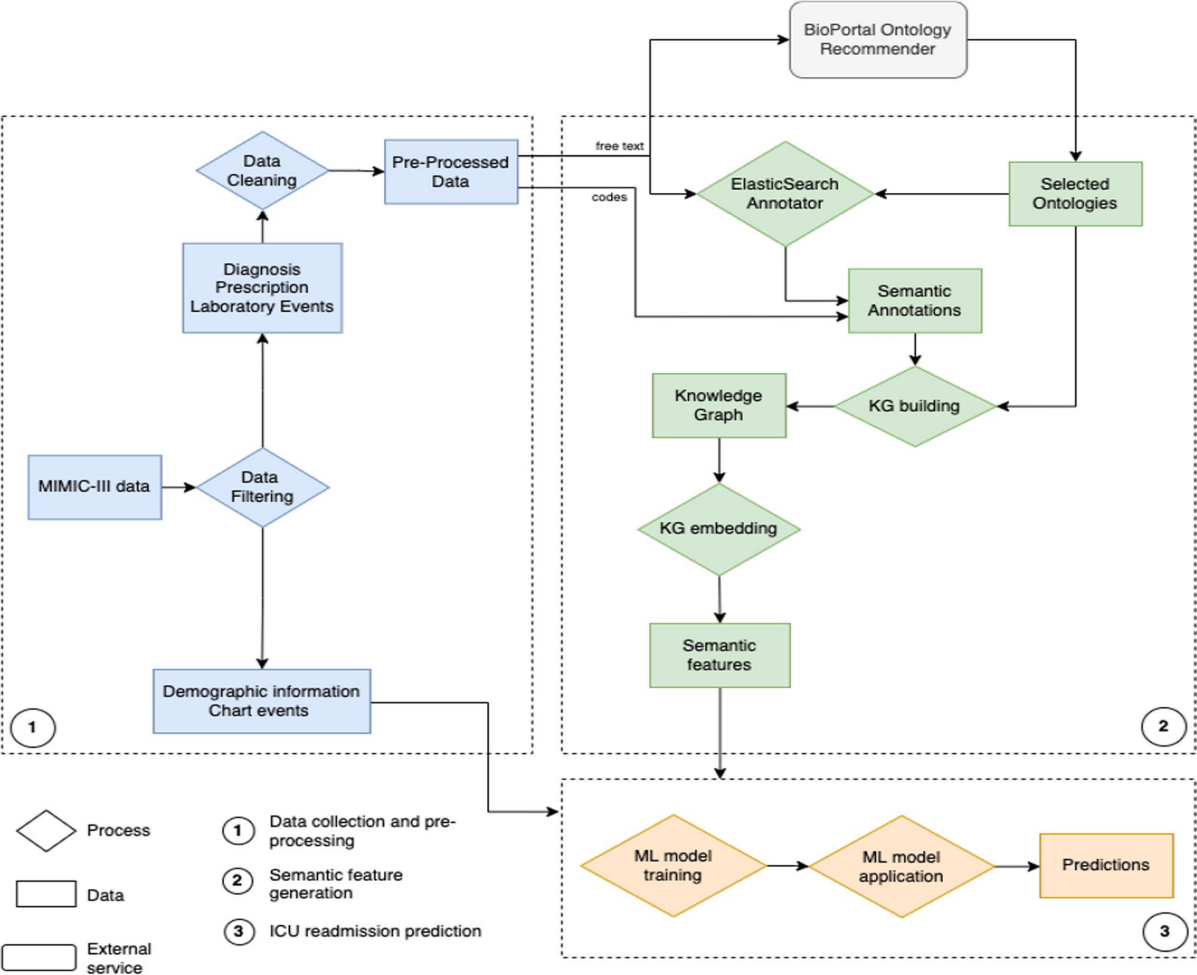
Overview of the methodology

An overview of the methodology is shown in Fig. 2. It has 3 main components: (1) data collection and pre-processing, (2) semantic feature generation, (3) readmission prediction. The data collection gathers all the ICU information needed for the ML models and semantic enrichment from the MIMIC-III database. The semantic feature generation step builds the KG and generates vector representations (i.e., embeddings) that can be processed by the ML models for prediction. The process takes as input the MIMIC-III dataset and a repository of biomedical ontologies (via BioPortal), and outputs a prediction for if a patient will be readmitted into the ICU in the 30 days following their release.

To better elucidate the impact of using semantic annotations and KG embeddings for ICU readmission prediction, we build on the work of Lin et al. [16], the only related work with open source code (see Availability of data and materials). Our methodology differs from theirs by both considering additional relevant information from the MIMIC-III dataset and by enriching this information with semantic representations of features based on ontology embeddings. While Lin et al. built a readmission prediction model based on three specific categories of features in the MIMIC-III data set, namely, chart events, ICD9 final diagnosis and demographic information for each patient, this work also includes prescriptions, initial diagnosis, procedures information and laboratory events. Lin et al. employed pre-trained ICD9 embeddings based on medical texts to represent the final diagnosis. We do not use them, and instead use KG embeddings to represent the semantic annotations for all features. Moreover, they are limited to predictions at the end of an ICU stay, since final diagnosis is only available then, whereas this work supports predictions at different moments of the ICU stay as more data becomes available. Finally, while Lin et al. employ sophisticated combinations of LSTM and CNN, we focus on more traditional ML algorithms to better discriminate the impact of semantic annotations from that of the choice of ML algorithm.

### Data collection and pre-processing

MIMIC-III [31] is an extensive, freely available database possessing records related to 53, 423 distinct hospital admissions of adult patients (aged 16 years or above) who stayed in the Beth Israel Deaconess Medical Center intensive care units between 2001 and 2012 [32]. The MIMIC-III database contains de-identified and comprehensive health-related intensive care data. It comprises relevant information such as demographics data, vital sign measurements, diagnosis, caregiver notes, procedures endured on the stay, laboratory tests and findings, prescriptions, and mortality.

#### Data acquisition and filtering

Four features were extracted from the MIMIC-III data set:

- *Patients’ demographics* extracted are age, gender, ethnicity and insurance type, following [16].
- *Chart events* are 17 features including notes, laboratory tests, fluid balance, etc., for each patient and normal median values extracted following [16].
- *Prescriptions* include the drug name and National Drug Code (NDC) code (not considered in [16]).
- *Diagnosis* includes two features: initial diagnosis, recorded at admission in free text (not considered in [16]), and final diagnosis coded in ICD9 at discharge with a matching label.
- *Procedures* are coded as $\text {ICD9}$ procedures (not considered in [16]).
- *Laboratory Tests* are coded using Logical Observation Identifier Names and Codes (LOINC), we extract the label and code as features (not considered in [16]). We do not use other data such as value, unit, etc.

**Fig. 3:**
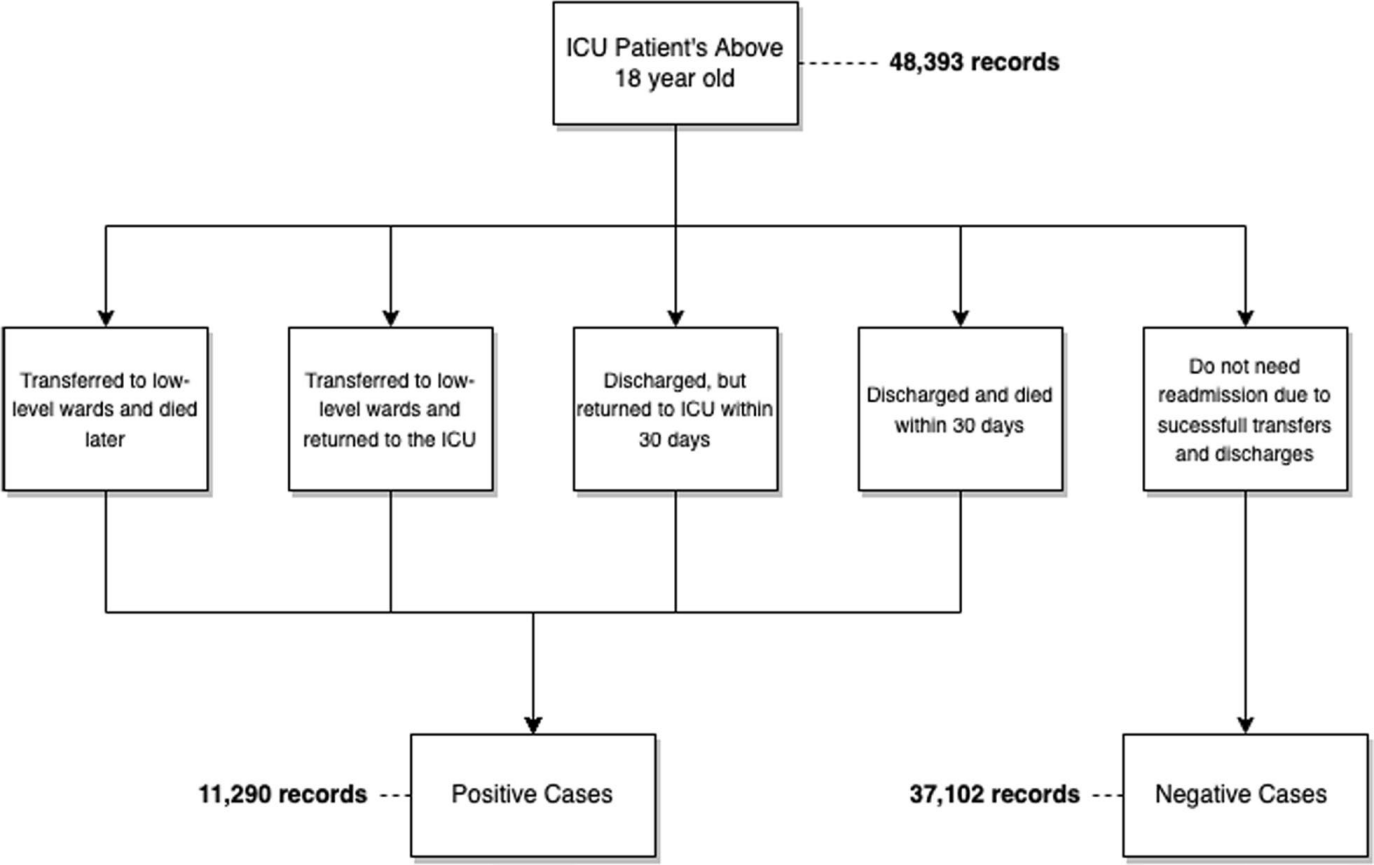
Patient’s records distribution according to the selection criteria on Lin et al. [16]

Following Lin et al. [16] we remove patients under the age of 18 years old, resulting in a total of 35,334 patients with 48,393 ICU stays. Figure 3 represents the patient classification into negative and positive instances. According to the criteria for patient’s selection, the following cases are considered to be ICU readmissions [16]: (1) The patients that were transferred to low-level wards from ICU, but returned to ICU again; (2) The patients that were transferred to low-level wards from ICU, and died later; (3) The patients that were discharged, but returned to the ICU within the next 30 days; (4) The patients that were discharged and died within the next 30 days. This results in a balance of 3:1 between records without readmission (negative) and records with readmission (positive). The total is 37,102 negative records and 11,290 positive records.

#### Data cleaning and pre-processing

For initial and final diagnosis, we exclude entries that have missing labels or labels containing unreadable characters. For the prescriptions, lab events and procedures, the features correspond to controlled vocabularies codes. However, there are still issues of missing data or codes in the wrong format. Entries with missing labels are excluded. Codes in the wrong format occur only in the International Classification of Diseases, Version 9-Clinical Modification (ICD9CM) controlled vocabulary. The MIMIC-III does not include the period character that is a part of ICD9CM codes, which creates ambiguity for the annotators between codes from different branches. Since MIMIC-III distinguishes between codes used for procedures and codes for diagnosis, we were able to reconstruct correctly formatted codes since procedures include the period after the second character, and diagnosis after the third.

#### ICU timeline snapshot split

An ICU stay has multiple stages from the moment a patient enters the unit, undergoes diagnosis exams and procedures, receives care, all leading ideally to a successful discharge and recovery. This means that throughout a patient’s ICU stay new information is generated, as drugs are prescribed, tests are prescribed and done, or procedures are performed.

**Fig. 4:**
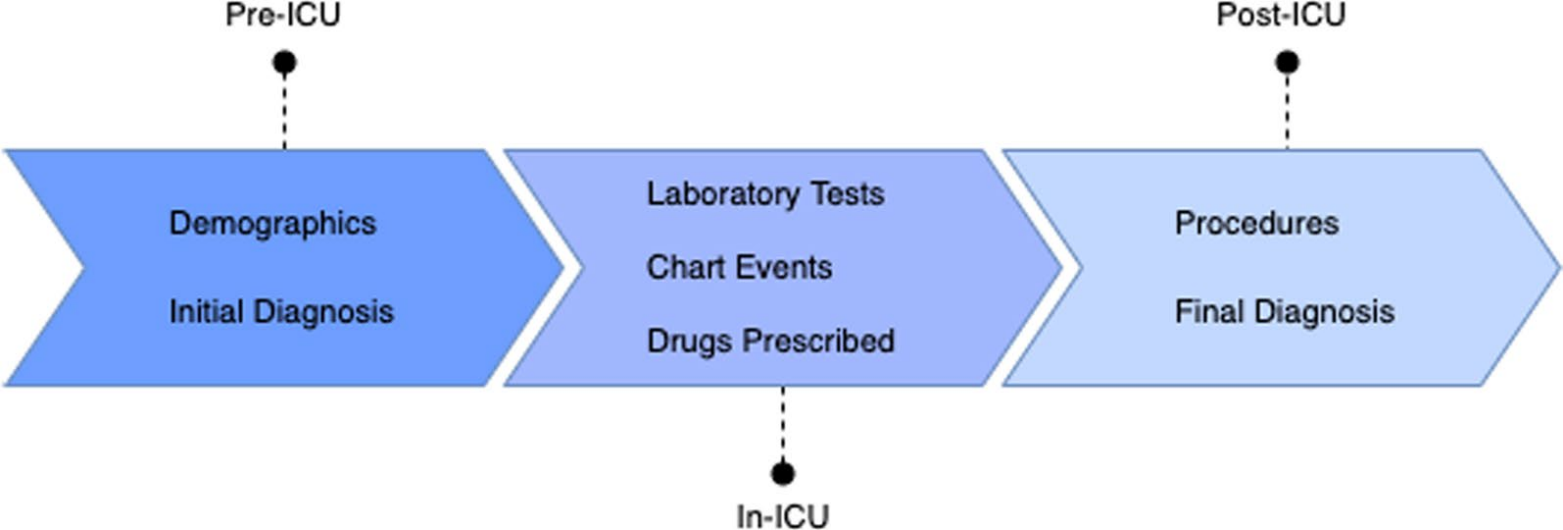
Prediction moments during an ICU stay with corresponding information

To capture the evolution of an ICU stay we consider three moments (snapshots) for which to make predictions: Pre-ICU, In-ICU and Post-ICU [33]. Figure 4 represents this timeline and the information that is available for each moment. Pre-ICU corresponds to the data available when the patient enters the ICU: demographic information and initial diagnosis. In-ICU includes Pre-ICU data as well as laboratory tests, prescribed drugs and chart events. Post-ICU includes all previous information as well as the information that is recorded at discharge: final diagnosis and procedures. Although procedures correspond to the In-ICU moment, since they are only recorded in the MIMIC-III EHR for billing purposes at the end of the stay we only include them in the final moment.

### Semantic feature generation

An ontology provides a specification of the meaning of the concepts in a domain and an associated vocabulary [34]. This specification means the context and the semantic rules that apply to concepts, allowing for their interpretation through their logical axioms. Using ontologies to represent domains reduces ambiguity and facilitates machine understanding. Clinical text is rich in synonyms, contributing to a high degree of ambiguity in analysis. Ontologies define multiple synonyms to represent the same concept and afford precise semantics for each concept, allowing the identification of synonyms in the text they annotate.

Ontologies serve as the semantic layer of smart information systems or more recently, as the schema layer of KGs [35]. When a substantial amount of data instances are structured in a graph, where nodes represent instances and edges the relations between them, and that graph follows a schema provided by an ontology to define the classes and relations of the instances, we can call it a KG.

The knowledge that ontologies (and controlled vocabularies) provide may be used in predictive models without prior data analysis or mining, to enrich or expand features, increasing the information available to the ML methods that would otherwise be unavailable [36]. This is especially true in the life science domain where there are more than nine-hundred ontologies available, spanning cross several fields of research on biological and biomedical domains [36]. Biomedical ontologies are able to provide controlled vocabularies for characterizing most biological phenomena with formalized domain descriptions and provide interaction by link them to other related domains [37].

We define a KG as a graph-representation of knowledge that describes entities and their relations defined according to classes and relations in an ontology. The approach to build a KG by linking the data extracted from the EHR to ontologies and using it to generate features includes three steps (see Fig. 2 step 2): (1) *Ontology Selection*, where ontologies that provide adequate coverage of the feature’s domains are selected; (2) *Semantic Annotation*, where textual features are mapped to ontology classes that describe them; and (3) *Annotation Embedding*, where each feature’s annotation is processed using a KG embedding approach that represents it in a numerical vector that reflects the meaning of the particular class within the ontology.

#### Ontology selection

The BioPortal Recommender platform [38] was used to support ontology selection. This service receives a biomedical text corpus or a list of keywords, for instance a set of EHR terms and for the set suggests ontologies appropriate for reference [38]. Despite the low number of studies describing the Bioportal recommender accuracy, we know it relies on the NCBO annotator for ontology annotations scoring and to provide a recommendation [39]. The annotator uses Mgrep for concept recognition [40], an extremely accurate system that for disease name recognition ensures a 95% or higher accuracy [40]. These high accuracies ensure that the bioportal recommender has a high accuracy for diagnosis annotation. We used a pre-selected group of ontologies of interest: National Cancer Institute Thesaurus (NCIT), Systematized Nomenclature of Medicine-Clinical Terms (SNOMEDCT), Medical Subject Headings Thesaurus (MeSH) and RxNORM were selected based on relevance attributed in a previous work [41]; LOINC, The Drug Ontology (DRON) and ICD9CM were selected due to their presence on the MIMIC-III data set; Medical Dictionary for Regulatory Activities Terminology (MedDRA) and Experimental Factor Ontology (EFO) were selected as extra relevant biomedical ontologies.

#### Semantic annotation

Semantic annotation is the process of describing an object by associating it with concepts that have well-defined semantics in an ontology [42]. Given the results obtained for the Ontology selection, we considered two annotation strategies: one using the NCIT and one using four different ontologies (NCIT, LOINC, ICD9CM and DRON).

For the single ontology scenario all textual labels (diagnoses, lab events,procedures and prescriptions) were mapped to a single ontology, NCIT.

For the multi-ontology scenario, the semantic annotation procedure is simpler because MIMIC-III already includes the codes (i.e., class identifiers) for the laboratory events (LOINC) and final diagnosis and procedures (ICD9CM). To cover the drug prescriptions, since NDC is not openly available, we mapped its classes to DRON using the BioPortal Annotator. Initial diagnoses were mapped via their textual labels to NCIT.

The text based annotations to NCIT were performed with ElasticSearch [43]. For each term in our dataset, a list of the six best scoring matched ontology classes is retrieved, and the one with the smallest Levenshtein Distance between the label of each class and the input term is selected.

A patient is thus initially represented by a vector of all their annotations, i.e. the ontology classes that describe their features.

1 $$\begin{aligned} P_{a}=\{c_1,\ldots , c_n\} \end{aligned}$$

The annotations are then used to build a KG that defines patients as instances that are related to the ontology classes that annotate them:

2 $$\begin{aligned} KG=\{V_c,V_i,E_c,E_a\} \end{aligned}$$

where $V_c$ are the vertexes that represent ontology classes, $V_i$ represent instances, $E_c$ the edges between classes, $E_a$ the edges between an instance and the class that annotates it.

#### KG embeddings

To represent patients through their semantic annotations for each feature in a way that machine learning algorithms can process, we employed KG embeddings. An embedding is a technique that transforms a higher dimensional space into a lower dimensional one [44]. KG embeddings represent the KG components in continuous vector spaces, so that their manipulation is simplified but at the same time preserve the inherent structure of the KG [45]. A typical KG embedding techniques has three steps [45]; the first specifies the entities and relations representation on the vector space, with entities usually represented as vectors and relations taken as operations represented as vectors or matrices; the second step defines a scoring function to measure the plausibility; and on the final step, to learn useful entity and relation representations, based on the score function, an optimization is done to maximize plausibility [45].

KG embeddings are learned for each annotated class of each ontology used, resulting in five sets of vector embeddings (one for the single ontology scenario one and four for the multi-ontology scenario two). The full graph is given as input to the KG embedding methods, with all types of relationships being considered. However, the majority of these are hierarchical relations, which is a consequence of the nature of the ontologies employed in our strategy.

We hypothesize that random-walk-based embedding techniques such as RDF2Vec are better suited to embedding instances based on their ontology annotations, because they are better at capturing long distance hierarchical relations than translational strategies like TransE [46]. Additionally, we also wanted to investigate whether methods that also take advantage of the ontology axioms and lexical component of the ontologies, such as OPA2Vec [22] would represent an improvement.

RDF2Vec [44] is a random-walk based strategy fit to handle specific semantics of RDF graphs (a language used to encode KGs and ontologies) [44]. For a given graph $G = (V, E)$, for every single vertex $v \in V$, RDF2Vec generates all graph walks $P_v$ of depth *d* rooted in the vertex *v*. These sequences are the input to *word2vec* [47], a two-layer neural net model to learn word embeddings from raw text (or in this case, sequences of graph entities).

OPA2Vec [22] produces a triple representation of the ontology based on formal axioms both materialized and inferred by reasoning and annotation axioms that capture the lexical component. It then applies a PubMed pre-trained Word2Vec model [48] to produce the embeddings vectors.

TransE [46] uses translations to represent relations in the embedding space, where for each entity and relation, if a triple of subject, predicate, and object (*s*, *p*, *o*) holds, the embedding of the object must be close to that of the subject plus a vector of the predicate (relation) [46]. This can than be generalized for every triple on the KG.

All embedding vectors have 300 dimensions, following the baseline embedding parameters used by Lin et al. [16] and after empirical evaluation of 200 and 400 dimensions showed no performance gain. Other parameters are set to default in both TransE and OPA2Vec. RDF2Vec employed the Skip-Gram algorithm, 500 walks and a maximum depth of 4. An ontology class is now represented as a vector with 300 dimensions.

If an ICU stay of a patient is annotated by more than one class (vector) within an ontology, then the vectors for each annotated class are summed. This aggregation approach follows the one used by [16] for the ICD9 embeddings. More formally, the embedding vector that represents a patient *p* under a given ontology *o* is given by the sum of each embedding vector $v_c$ that represents each annotation of the patient in *o* to a class *c*.

3 $$\begin{aligned} v_{p_o} = \sum _{c=1}^{n} v_c \end{aligned}$$

Since in the multi-ontology scenario each ICU stay feature is annotated by a different ontology, this results in four different embeddings vectors each corresponding to the sum of the individual vectors for each annotation. These four vectors are then concatenated (i.e., appended) instead of summed to preserve the distinct dimensions.

This results in a single vector describing an ICU stay of a patient with 300 dimensions for the single ontology scenario and 1200 dimensions for the multi-ontology scenario.

### Readmission prediction

The prediction task is formulated to correctly predict if a patient will be readmitted to an ICU unit within 30-days after release (or die). Each instance corresponds to a patient and their ICU stay, represented by a concatenated vector that includes demographic data ($v_d$) and chart events ($v_c$) (similarly to [16]) as well as the KG embeddings vector ($v_{p_o}$):

4 $$\begin{aligned} P =\{v_{d} + v_{c} + v_{p_o}\} \end{aligned}$$

Predictions at different points of the ICU timeline stay include only the embeddings for the data available at that time.

Four classical machine learning methods are used: Logistic Regression (LR), Random Forest (RF), Naive Bayes (NB), and Support Vector Machine (SVM). These are the same methods used by [16] as baseline models. The LSTM and CNN models were not reproducible, possibly due to an incompatibility of libraries. No hyperparameter optimization is applied, to ensure a more direct comparison to [16]. The choice of using classical methods allowed us to focus our analysis on the impact of the KG embeddings.

## Results and discussion

### Data cleaning results

**Table 1:** Number of terms for each feature type before and after the cleaning process, and after semantic annotation

	Initial diagnosis	Lab tests	Drug prescriptions	Procedures & Final diagnosis	Total
Original data	3567	574	2782	7993	14916
Cleaned Data	2709	568	2782	6678	12737
Multi ontology annotated terms	2709	568	2782	6678	12737
NCIT annotated terms	2709	263	856	1118	4946

737 patients ( 2%) in the Pre-ICU setting do not have an initial diagnosis and were removed from that experiment, but all patients are retained for both In-ICU and Post-ICU, since they all have at least one initial diagnosis, drug prescription or lab test.

After the data cleaning procedures there are 12,737 different unique terms that can be annotated (see Table 1), representing a total of 85.4% of the original set. The losses were as expected more pronounced for the *Initial Diagnosis* because as a free text variable it is more likely to suffer from issues such as meaningless characters (e.g., a single dash or period instead of alphabetical characters).

**Fig. 5:**
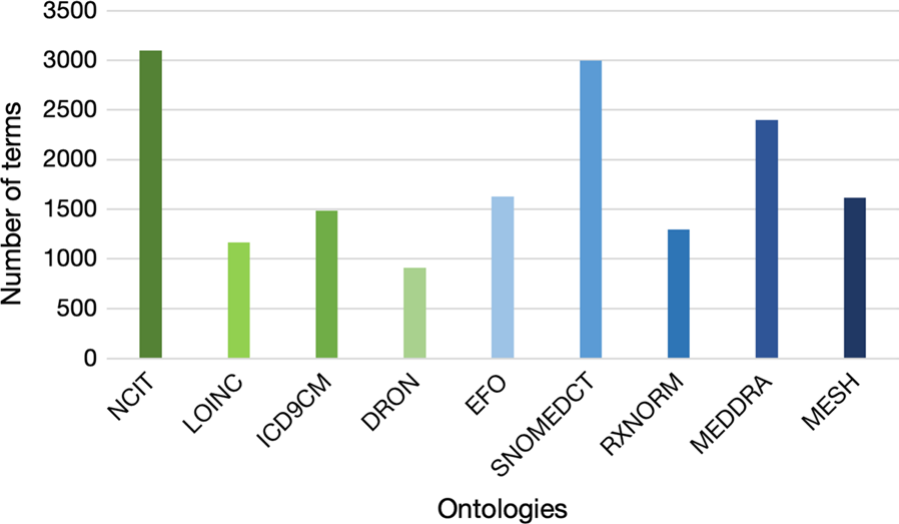
Number of terms annotated with each individual ontology proposed by BioPortal

### Semantic feature generation results

After running the BioPortal recommender for all terms extracted from our dataset, the NCIT ontology is the most suitable (Fig. 5), with the best overall coverage reaching about 35% coverage. Although NCIT is the best ontology for this prediction scenario, the MIMIC-III data set has mappings to specific ontologies according to the type of feature, LOINC for laboratory events, ICD9CM for procedures and final diagnosis, and NDC for drug prescriptions, which can also be used for annotation.

Table 1 presents the number of terms that served as input for annotation from each type (row *Cleaned Data*) and how many were annotated in the NCIT only and Multi-Ontology scenarios. The Multi-ontology annotation provides annotations for all input terms. However, the NCIT annotation results in a loss of information that is more pronounced for *Drug Prescriptions* and *Procedures* and *Final Diagnosis* since NCIT does not cover these aspects in as much detail.

The semantic annotations were used to build two KGs, one for each ontology scenarios. The resulting number of triples ((*subject*, *predicate*, *object*) statements) is presented on Table 2.

**Table 2:** KG statistics

	Triples	Subjects	Objects	Predicates
One ontology	6921258	519583	607819	83
Multiple ontologies	8288250	519583	618193	83

### Experimental design

The experimental design includes different components:

- *Reproduction results* a reproduction of the baselines established by [16].
- *NCIT embeddings for diagnosis* an evaluation of the impact of considering NCIT RDF2Vec embeddings for initial diagnosis information, coupled with the demographic information and chart events.
- *NCIT embeddings for all features* an evaluation of the impact of considering embeddings for all the features we extracted. Each feature set is represented by an NCIT RDF2Vec embedding vector.
- *Embeddings using multiple ontologies* a comparison of using embeddings based on different ontologies: NCIT for the initial diagnosis and the MIMIC-III proposed ontologies for each respective feature type. Each feature set is represented by an RDF2Vec embedding made with the specific ontology.
- *ICU stay simulation* a simulation of an ICU stay. Predictions are made for each of the three moments where new information becomes available (Fig. 4).
- *Other KG embeddings approaches* a comparison of the best performing model using representative methods of distinct KG embeddings approaches.
- *Ablation study* an assessment on the impact the KG completeness has on predictive performance.

We performed a five-fold cross-validation and measured the AUROC to assess the trade-off between sensitivity and specificity and AUPRC to better analyse the performance over the positive instances without having to establish a threshold. We also performed statistical Kruskal–Wallis tests over the AUROC and AUPRC values to evaluate the significance of the results. To illustrate the results, we also present Receiver Operating Characteristic (ROC) and Precision-Recall Curve (PRC) for single folds.

### Reproduction results

**Fig. 6:**
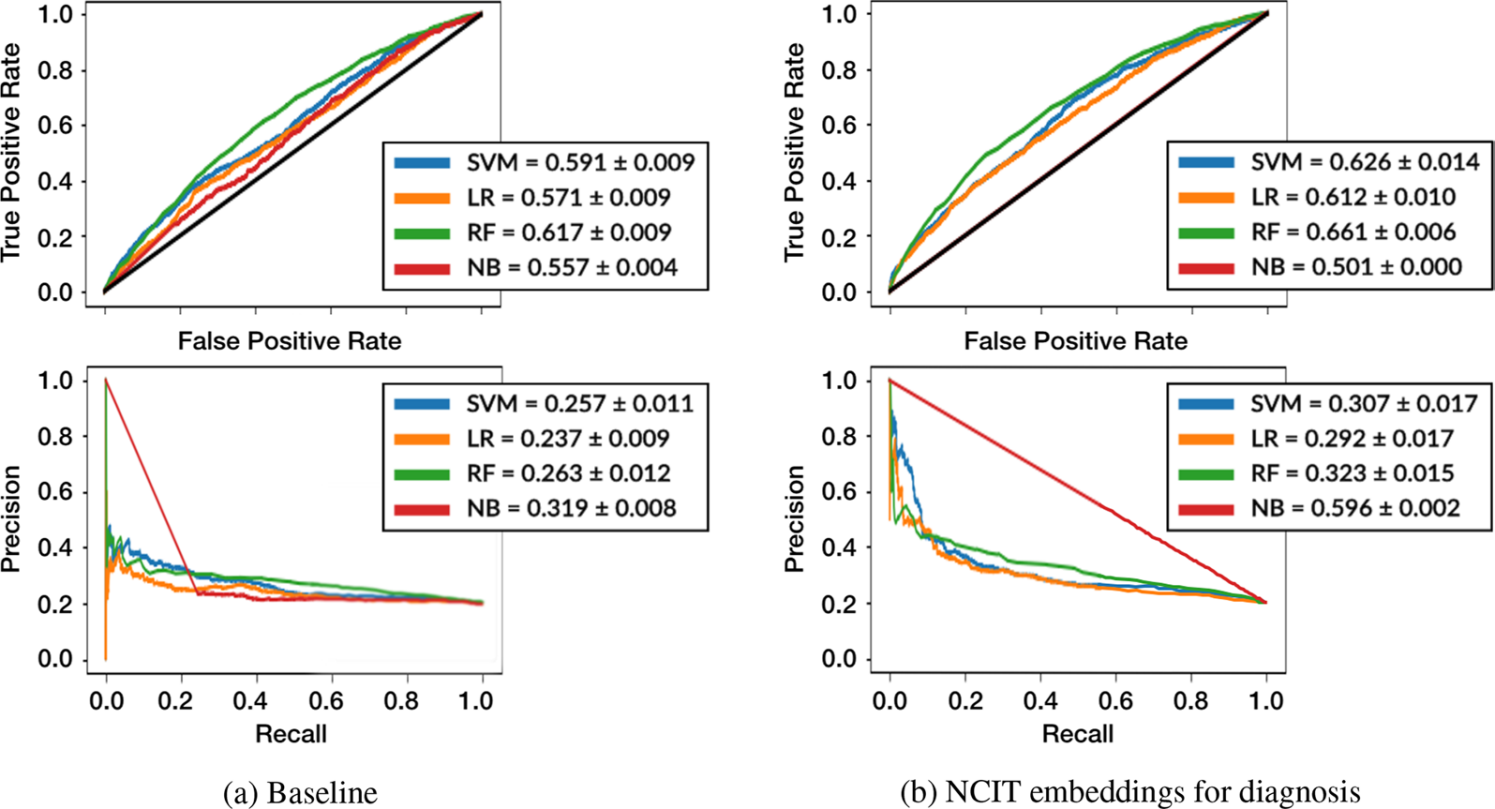
ROC and PRC comparison of the baseline and using NCIT embeddings for diagnosis instead of ICD9 embeddings. The boxes present mean and standard deviation of AUROC and AUPRC for the four ML algorithms

The reproduction results on Fig. 6a are somewhat lower than the ones reported, likely due to differences in the cleaning and pre-processing steps. The results obtained were mean AUROC values of 0.591 $\pm 0.009$ for SVM, 0.571 $\pm 0.009$ for LR, 0.617 $\pm 0.009$ for RF and 0.557 $\pm 0.004$ for NB, meaning that all four prediction models are relatively poor in terms of performance. Mean AUPRC values are also low (0.257 $\pm 0.011$ for SVM, 0.237 $\pm 0.009$ for LR, 0.263 $\pm 0.012$ for RF and 0.319 $\pm 0.008$ for NB).

### NCIT embeddings for diagnosis

The performance of the NCIT Embeddings for diagnosis approach (see Fig. 6b) slightly improves on the reproduction results, with RF achieving the best result with an AUROC of 0.661 $\pm 0.006$, and all methods above 0.610 except $\text {NB}$. The AUPRC is also increased compared to the baseline. This indicates that there is valuable information in the initial diagnosis, which when semantically enriched is able to outperform the baseline that has access to the final diagnosis, which one could hypothesize has better predictive value for the readmission risk.

The Kruskal–Wallis analysis between the baseline and NCIT diagnosis embeddings resulted in p-values for the AUROC analysis ranging from 0.009 to 0.016, and from 0.008 to 0.009 for the AUPRC, which further indicates that NCIT initial diagnosis embeddings represent an improvement over the ICD-9 final diagnosis embeddings.

### NCIT embeddings for all features

**Fig. 7:**
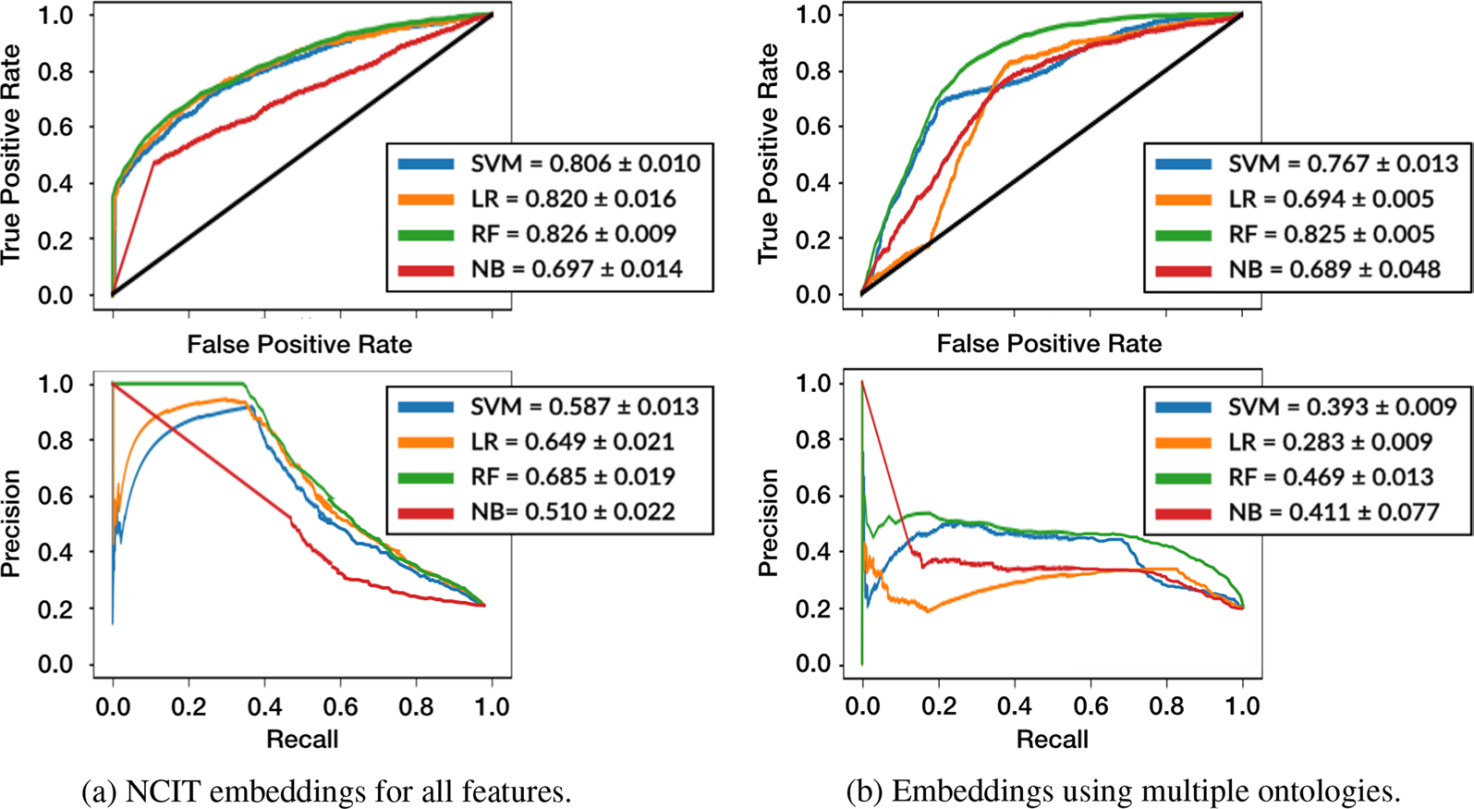
Comparison of $\text {ROC}$ and $\text {PR}$ curves with AUROC and AUPRC (mean and standard deviation values) for the two semantic annotation strategies: using one or multiple ontologies

This experiment targeted the prediction of readmission at the end of the ICU stay and used all information collected and annotated with NCIT (see Fig. 7a).

Performance improved not only over the reproduction baselines but also significantly over the previous experiment, with RF as the best performing method achieving mean AUROC of 0.826 $\pm 0.009$ and all models achieving values above 0.8, with exception of the NB model. However, the largest improvement was on the AUPRC where the trade-off between precision and recall is now improved with the best performing model achieving a 0.685 $\pm 0.019$ mean AUPRC value. These results clearly showcase the benefit of considering semantically-enriched information throughout the ICU stay.

The statistical analysis produced p-values between 0.008 and 0.009 for both AUROC and AUPRC when comparing both against the baseline and the NCIT embeddings for diagnosis. These results further support the significance of the observed improvements in both cases.

### Embeddings using multiple ontologies

The results of the multiple ontologies embeddings experiment on Fig. 7b show that overall the performance decreases when compared to using only NCIT. Although RF presents a mean AUROC value of 0.825 $\pm 0.005$, a clear improvement over the corresponding baseline (0.617 $\pm 0.009$), the rest of the models under-perform when compared to the single ontology approach. The AUPRC results also highlight the decrease in performance, with all the models performing under 0.5. These results demonstrate that employing multiple ontologies tailored to different domains is not a better approach over using a single general purpose ontology. This is likely due to the fact that learning embeddings in a single semantic space that can be readily combined afford a more holistic representation than training separate embeddings for each feature type using different ontologies. We hypothesize that this holistic representation can be better explored by the ML methods, and thus achieve better performance.

The Kruskal–Wallis test comparing the one ontology approach with the multiple ontologies approach revealed that for SVM and LR both AUROC, and AUPRC have low p-values ($\approx 0.009$), whereas RF and NB display values above 0.05. These results support that there is not a clear advantage to using specialized ontologies.

### ICU timeline snapshot prediction

**Fig. 8:**
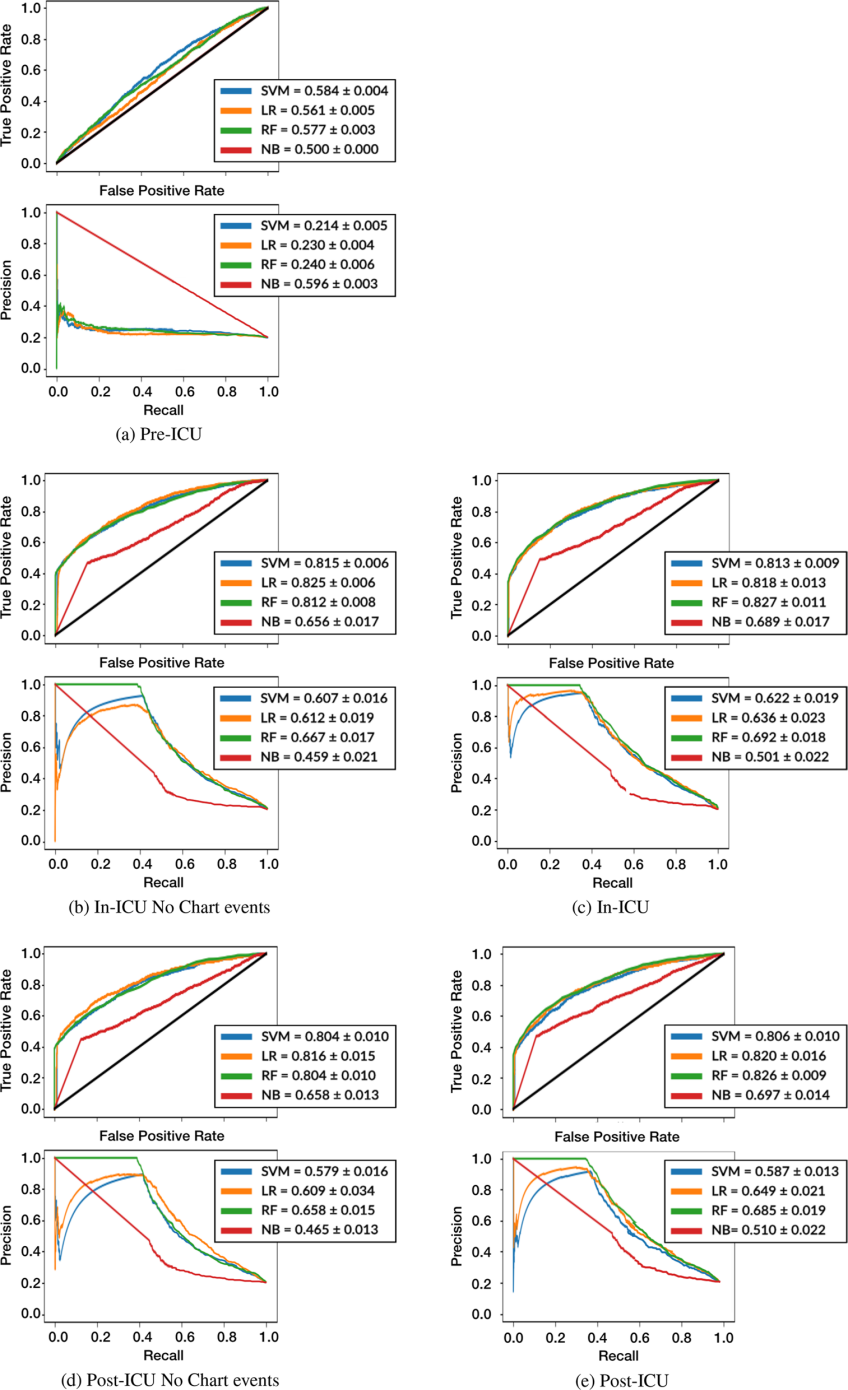
Comparison of ROC and PR curves with AUROC and AUPRC (mean and standard deviation values) for the different stages of an ICU [33], with the data mapped to the NCIT ontology, with and without chart events

To investigate prediction performance at the three ICU stay moments, pre-ICU, in-ICU and post-ICU, we use the best overall performing approach: all the information mapped to the NCIT ontology. The results obtained for this experiment are shown in Fig. 8.

The *Pre-ICU* approach is equivalent to the first experiment but without chart events. The results reveal that the improvement observed in the first experiment is due to an interplay between the initial diagnosis and chart events. However, since chart events are unavailable at the moment of admission, the initial diagnosis proves to be insufficient to achieve acceptable readmission prediction. The statistical analysis results in p-values below 0.05 for the AUROC and for the AUPRC) for all ML methods except NB.

During the stay, more information becomes available and the *In-ICU* experiment corresponds to all information gathered up to the discharge event. Here, we observe the largest performance improvement with AUROC values for RF achieving 0.827 $\pm 0.011$ and AUPRC 0.692 $\pm 0.018$, when all data is considered. The statistical test comparing Pre-ICU and In-ICU produces p-values below 0.009 for AUROC and AUPRC, supporting the conclusion that the additional information captured during the ICU stay contributes to a better predictive performance.

When chart events are not considered, performance is only slightly decreased, indicating that it is indeed the laboratory information and drug prescriptions embeddings that positively impact performance. In fact, none of the statistical achieve p-values below 0.05.

Finally, at *Post-ICU* we observe a slight decrease in mean performance both in AUROC and AUPRC. This is a curious effect, which indicates that final diagnosis and procedures do not have an additive contribution to ICU readmission prediction.

The results indicate that it is possible to predict ICU readmission with good performance with the information that is collected during an ICU stay, without needing the final diagnosis and procedures. The confusion matrix in Table 3 shows a high average true negative rate clearly showing that the In-ICU model is capable of preventing the unnecessary readmission of more than 90% of the patients. Additionally, for about 55% of patients who need to stay in the hospital, the model can prevent early discharge. It is especially interesting to note that these are carried out while just unnecessarily prolonging the stay of 10% of the patients who would not require it.

**Table 3:** Confusion matrix of average RF In-ICU test fold records distribution

	Predicted negative	Predicted positive
Actual negative	3400	510
Actual positive	380	600

### Other KG embedding methods

**Fig. 9:**
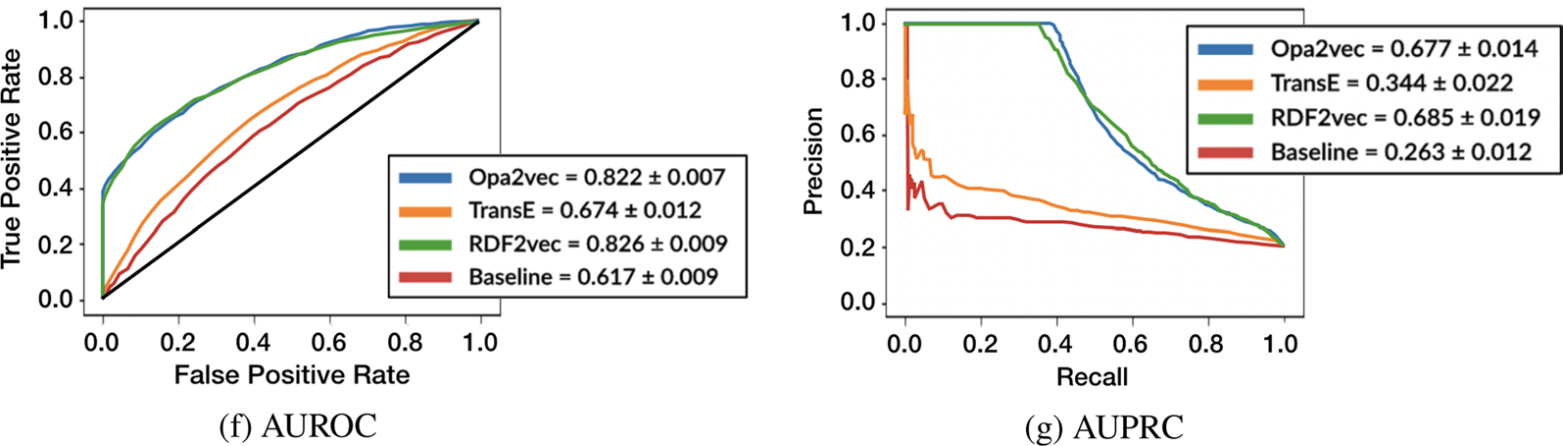
Comparison of KG embedding techniques coupled with RF using the best performing strategy (*In ICU* with NCIT ontology)

The TransE embeddings outperform the baseline, but do worse than both OPA2Vec and RDF2Vec, which confirms our initial hypothesis (see Fig. 9). OPA2Vec achieves much better performance with mean AUROC of 0.822 $\pm 0.007$ and AUPRC of 0.677 $\pm 0.014$. Although these values are somewhat lower than those achieved by RDF2Vec, the Kruskal–Wallis test produced values well above 0.05 (0.590 and 0.207 for AUROC and AUPRC respectively), indicating no significant differences in the performance of both methods. One can argue that whatever advantages OPA2Vec gains by considering the lexical portion of the ontology, are compensated by RDF2Vec’s long random walks over the graph.

### Ablation study

**Fig. 10:**
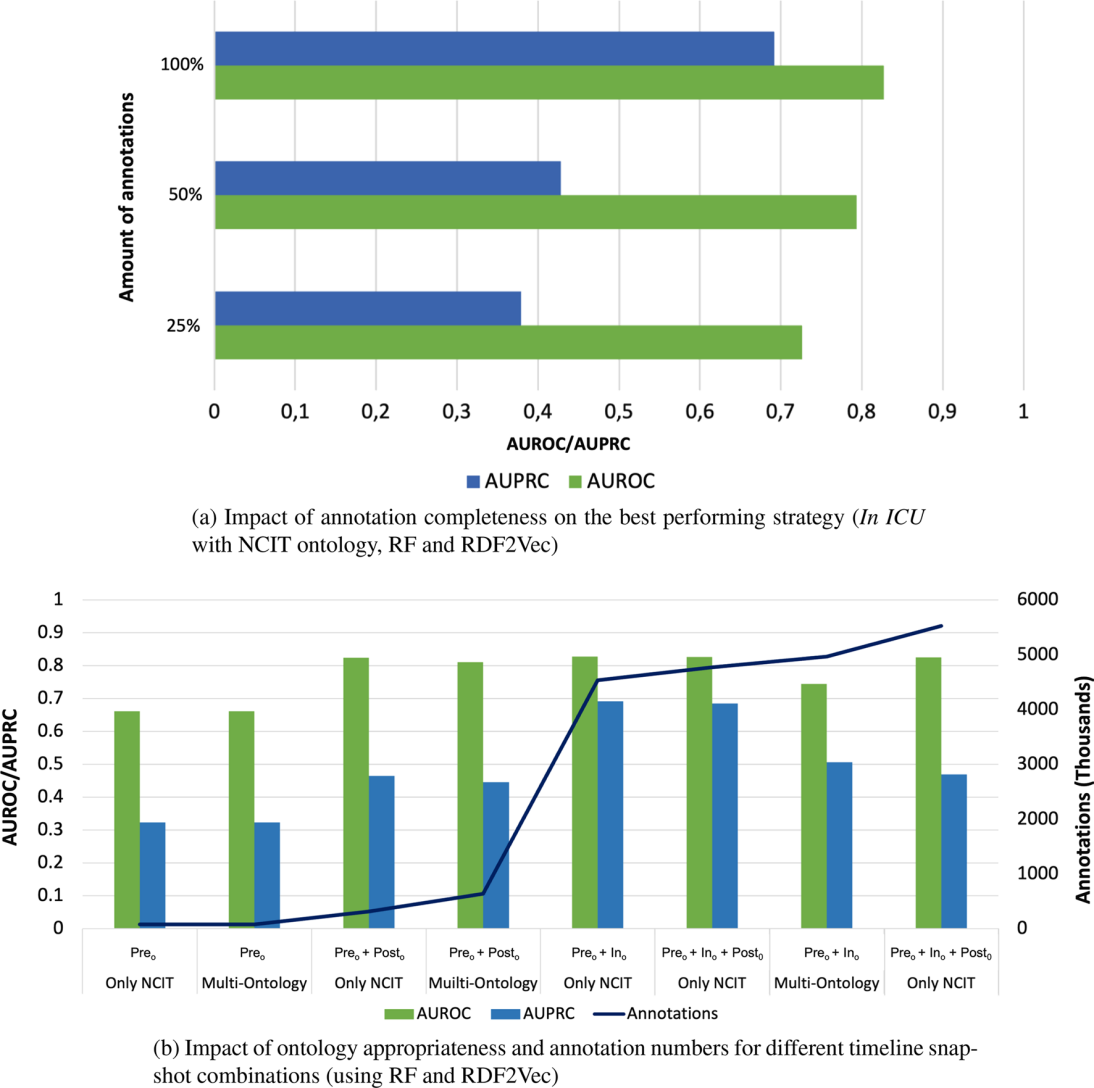
Evaluation of the impact of KG completeness

We designed further experiments to assess how the KG completeness, in terms of the number of annotations and coverage of the ontologies impacts predictive performance. Although MIMIC-III contains real data and thus represents the completeness of data achieved at one real hospital, data completeness is expected to vary considerably between different institutions.

Figure 10a showcases the effect that randomly reducing the number of annotations for each patient down to 50 and 25% has on performance. The impact is more noticeable in terms of AUPRC, but AUROC remains above 0.7 even with just 25% of the annotations available. Figure 10b supports a global analysis of all annotations available across the different scenarios and ontology sets. For this experiment, we did not use the cumulative annotations for each moment, but rather used the annotations available only for a specific moment and composed them into different combinations. This allows us to test the scenario where we have the *Pre-ICU* and *Post-ICU* annotations but not the *In-ICU*. These experiments show that in general performance grows with annotation numbers as expected, however the best overall performance is actually achieved for the $Pre_o + In_o$ scenario where fewer annotations are available compared to $Pre_o + In_o + Post_o$, and that the multi-ontology scenario performs worse than the single-ontology scenario despite having a more comprehensive KG and more annotations. Furthermore, the lower number of annotations present at $Pre_o + Post_o$ are capable of achieving a good performance in terms of AUROC. These results highlight that although the completeness of the data does have an impact on predictive performance, not all annotations are created equal and that performance is influenced by the interplay between annotation number, type of information covered by the annotations, and ontologies used.

### Comparison with the state of the art

Although this work has several parallels to Lin et al. [16] since they use the same dataset and target the same prediction task, both works differ substantially in the features they employ, how they generate those features and the ML algorithms applied. The work by Lin et al. has a strong focus on chart-events, which are the main input to their recurrent neural network model, whereas in our case chart events do not actually impact predictions made by our models. Our experiments revealed that predictions made with or without chart events result in the same performance for our method. They use fewer non-numerical features, extracting only demographics and the ICD9 final diagnosis codes, whereas in our work we consider initial diagnosis (text), laboratory events, prescriptions and final diagnosis (codes). To tackle the difficulties in comparing the ICD9 codes taking into account their meaning, Lin et al. use pre-trained word embeddings for ICD9 codes that were trained on a dataset of clinical notes. Our work, on the other hand, not only represents a lot more textual and categorical features, but it does so by employing embeddings based on biomedical ontologies. The KG embeddings allow us to tackle a considerable challenge faced by EHR mining approaches: how to adequately compare categorical and textual features. The embeddings not only allow for a representation that can be explored by ML algorithms, but also, and perhaps more importantly they allow us to tap sources of information in the EHR that would otherwise not be accessible. A direct comparison between the performance obtained in the two works is not feasible, however, since we reproduced their baseline, we can compare the improvement they achieved over their baseline, to the improvement we obtained over our corresponding baseline. Lin et al.’s AUROC for the baseline using RF was 0.712 (ours was 0.617 $\pm 0.009$), whereas their best result (using LSTM + CNN) was 0.791 (ours was 0.827 $\pm 0.011$). The marked improvement our approach achieved clearly supports the advantages of scientific data contextualization.

Lu et al. [17] also targeted ICU readmission prediction, but employed text mining approaches over the text of discharge summaries. They reported an AUROC of 0.825 (our best approach, NCIT + RF, reached 0.827 $\pm 0.011$) and a AUPRC of 0.632 (our best approach, NCIT + RF, reached 0.692 $\pm 0.018$). The results are not directly comparable to ours in purely methodological terms, but they highlight that considering more features and enriching them with semantic annotations can have a positive impact of ICU readmission risk prediction.

## Conclusion

The growing adoption of EHR and the recent developments in ML applied to clinical data present an opportunity to address ICU readmission by generating accurate risk predictions that can help to reduce the number of readmissions and improve health outcomes. Clinical data has a rich background knowledge, but this is not accessible in EHRs and typical ML approaches are unable to explore it. We have developed an approach that enriches EHR data with semantic annotations to ontologies, and then generates KG embeddings to represent patient’s features in a contextualized manner.

We evaluated our approach in the MIMIC-III EHR data set, and experimented with different ontologies and controlled vocabularies (NCIT, LOINC, DRON and ICD9CM), KG embeddings techniques and ML algorithms. The best results were obtained using RDF2Vec embeddings of the NCIT ontology coupled with a Random Forest, achieving a mean AUROC of 0.827 $\pm 0.011$ and AUPRC of 0.692 $\pm 0.018$. These results represent a gain in more than 0.2 in AUROC and 0.4 in AUPRC over the baseline. We also experimented with making predictions at different moments of the ICU stay and with different levels of annotation completeness, and learned that the maximum predictive power is achieved without considering information only available at the moment of discharge. These results highlight that performance is influenced by data completeness but also by data domain and ontology appropriateness.

The general methodology developed in this work can be generalized to other clinical data sets and even other predictive targets, as long as the data therein is adequately covered by existing ontologies. Given the abundance and diversity of biomedical ontologies available, adequate coverage is highly likely for most applications. This work focused on classical ML approaches, given the small size of the data. However, there is also an opportunity to explore KGs using deep learning approaches, especially for larger datasets and a greater amount of features. A particular avenue for future work lies in exploring deep learning methods that are able to explore the temporal aspects of the features (e.g. [49]), and couple this with their semantic representations.

The coupling of semantic annotation and KG embeddings affords two clear advantages for ML applications in EHR: they consider scientific context by using ontology annotations that can then be explored by KG embeddings methods; they are able to build representations of EHR information of different types in a common format, since embeddings can represent any number of diagnosis, tests, procedures, etc, in a numerical vector that is easily processed by ML methods.

This work demonstrates the potential for impact that integrating ontologies and KGs into biomedical machine learning applications can have. Moreover, by having the clinical data semantically annotated with ontologies, this work also paves the way for more explainable approaches that explore the meaning encoded in ontologies to better explain predictions to clinicians [50, 51].

## Data Availability

The raw clinical MIMIC-III information is publicly available on PhysioNet: https://physionet.org/content/mimiciii/1.4/. The public MIMIC-III code is available at: https://doi.org/10.5281/zenodo.821872. The patient’s files needed for the implementation, can be obtained following the implementation on the [16] GitHub page: https://github.com/Jeffreylin0925/MIMIC-III_ICU_Readmission_Analysis. The complementary enriched files and embeddings we produced for the new implementation can be found on our GitHub page: https://github.com/liseda-lab/MIMIC-III-Enriched.

## Abbreviations

ICU: Intensive Care Unit
ROC: Receiver Operating Characteristic
PR: Precision-Recall
EHR: Electronic Health Records
MedDRA: Medical dictionary for regulatory activities terminology
ML: Machine learning
SVM: Support Vector Machine
AUROC: Area Under the Receiver Operating Characteristic Curve
LR: Logistic Regression
RF: Random Forest
NB: Naive Bayes
PRC: Precision-Recall Curve
AUPRC: Area Under the Precision-Recall Curve
NCIT: National Cancer Institute Thesaurus
ICD9CM: International Classification of Diseases, Version 9-Clinical Modification
ICD9: International Classification of Diseases, Version 9
LOINC: Logical Observation Identifier Names and Codes
NDC: National Drug Code
DRON: The Drug Ontology
KG: Knowledge Graph
CNN: Convolutional neural network
LSTM: Long short-term memory
SNOMEDCT: Systematized Nomenclature of Medicine-Clinical Terms
MeSH: Medical Subject Headings Thesaurus
EFO: Experimental factor ontology
